# Supplementary material for: Sexually transmitted infections and factors associated with risky sexual practices among female sex workers: A cross sectional study in a large Andean city
Source: PLoS One. 2021 May 6;16(5):e0250117. doi: 10.1371/journal.pone.0250117 (PMC8101946; doi:10.1371/journal.pone.0250117)
Supplement: S1 Table — (DOCX) [file pone.0250117.s001.docx]

**S1 Table.** **Gene targets and primers used for current diagnosed of STIs in this study**

| **Pathogen, target, primer and probe References** |
| --- |
| ***Neisseria gonorrhoeae***  opa genes (opa) [35s]  [NG_F](javascript:void(0)): TTGAAACACCGCCCGGAA  [NG_R](javascript:void(0)): TTTCGGCTCCTTATTCGGTTTGA  NG_P: [6FAM]CCGATATAATCCGCCCTTCAACATCAG[BHQ1]  ***Trichomonas vaginalis***  T. vaginalis‐specific repeat DNA fragment [36s]  TV_F: AAAGATGGGTGTTTTAAGCTAGATAAGG  TV_R: TCTGTGCCGTCTTCAAGTATGC  TV_P: [6FAM]AGTTCATGTCCTCTCCAAGCGTAAGT[BHQ1]  ***Chlamydia trachomatis***  The cryptic plasmid [37s]  [CT_F](javascript:void(0)): CATGAAAACTCGTTCCGAAATAGAA  [CT_R](javascript:void(0)): TCAGAGCTTTACCTAACAACGCATA  CT_P: [6FAM]TCGCATGCAAGATATCGA[MGBNFQ]  ***Mycoplasma genitalium***  MgPa adhesin gene [38s]  [MG_F](javascript:void(0)): GAGAAATACCTTGATGGTCAGCAA  [MG_R](javascript:void(0)): GTTAATATCATATAAAGCTCTACCGTTGTTATC  MG_P: [6FAM]ACTTTGCAATCAGAAGGT[MGBNFQ] |

**References**

[35s] Tabrizi SN, Chen S, Tapsall J, Garland SM. Evaluation of *opa*-based real time PCR for detection of *Neisseria gonorrhoeae*. *Sex Transm Dis*.2005; 32:199–202.

[36s] Pillay A, Radebe F, Feheler G, Htun Y, Ballard RC. Comparison of a TaqMan-based real-time polymerase chain reaction with conventional tests for the detection of *Trichomonas vaginalis*. *Sex Transm Infect*.2007;83:126–129.

[37s] Jaton K, Bille J, Greub G. A novel real-time PCR to detect *Chlamydia trachomatis* in first- void urine or genital swabs. *J Med Microbiol*.2006;55:1667–1674.

[38s] Jensen JS, Björnelius E, Dohn B, Lidbrink P. Use of TaqMan 5´ Nuclease Real-Time PCR for Quantitative Detection of *Mycoplasma genitalium* DNA in Males with and without Urethritis Who Were Attendees at a Sexually Transmitted Disease Clinic. *J Clin Microbiol*. 2004;42(2):683–692.
