# Supplementary material for: Sexually transmitted infections and factors associated with risky sexual practices among female sex workers: A cross sectional study in a large Andean city
Source: PLoS One. 2021 May 6;16(5):e0250117. doi: 10.1371/journal.pone.0250117 (PMC8101946; doi:10.1371/journal.pone.0250117)
Supplement: S1 File — (PDF) [file pone.0250117.s003.pdf]

Logistic Regression

Case Processing Summary

Unweighted cases[a]		N	Percent
Selected cases	Included in Analysis	249	100.0
	Missing cases	0	.0
	Total	249	100.0
Unselected cases		0	.0
Total		249	100.0

a. If weight is in effect, see classification table for the total number of cases.

Dependent variable Encoding

Original value	Internal value
No	0
Yes	1

Block 0: Beginning Block

Classification table[a,b]

			Predicted		
			RSP		Percentage correct
Observed			No	Yes	
Step 0	RSP	No	193	0	100.0
		Yes	56	0	.0
	Overall Porcentage				77.5

a. Constant is included in the model.

b. The cut value is .500

Variables in the Equation

		B	Standard error	Wald	df	Sig.	Exp(B)
Step 0	Constant	-1.237	.152	66.454	1	.000	.290

Variables not in the Equation

			Score	df	Sig.
Step 0	Variables	Age	26.050	1	.000
		Membership_FSWs_association(1)	22.386	1	.000
		Self_report_STI(1)	15.288	1	.000
		Currently_diagnosed(1)	4.580	1	.032
		Previous_treatment_STI(1)	.311	1	.577
		Temporary_migration_SW (1)	.063	1	.802
	Overall statistics		45.938	6	.000

Block 1: Method = Enter

Omnibus Tests of Model Coefficient

		Chi-square	df	Sig.
Step 1	Step	44.600	6	.000
	Block	44.600	6	.000
	Model	44.600	6	.000

Model Summary

Step	-2 Log likelihood	Cox & Snell R Square	Nagelkerke R Square
1	220.853[a]	.164	.250

a. Estimation terminated at iteration number 5 because parameters estimates changed by less than .001.

Classification Table[a]

Observed			Predicted: RSP No	Predicted: RSP Yes	Predicted: Percentage correct
Step 1	RSP	No	185	8	95.9
		Yes	40	16	28.6
	Overall Percentage				80.7

a. The cut value is .500

Variables in the Equation

		B	Standars error	Wald	df	Sig.
Step 1[a]	Age	.060	.019	9.755	1	.002
	Membership_FSWs_association(1)	1.256	.402	9.774	1	.002
	Self_report_STI(1)	1.233	.503	6.004	1	.014
	Currently_diagnosed(1)	.257	.418	.379	1	.538
	Previous_treatment_STI(1)	.222	.407	.297	1	.586
	Temporary_migration_SW (1)	.589	.398	2.190	1	.139
	Constant	-4.504	.857	27.630	1	.000

Variables in the Equation

			95% C.I. for EXP(B)	
		Exp(B)	lower	Upper
Step 1[a]	Age	1.062	1.023	1.102
	Membership_FSWs_association(1)	3.512	1.598	7.721
	Self_report_STI(1)	3.433	1.280	9.207
	Currently_diagnosed(1)	1.293	.570	2.935
	Previous_treatment_STI(1)	1.249	.562	2.775
	Temporary_migration_SW (1)	1.803	.826	3.935
	Constant	.011		

a. Variables entered on step 1: Age, Membership_FSWs_association, Self_report_STI, Currently_diagnosed, Previous_treatment_STI, Temporary_migration_SW.
