# Supplementary material for: Sexually transmitted infections and factors associated with risky sexual practices among female sex workers: A cross sectional study in a large Andean city
Source: PLoS One. 2021 May 6;16(5):e0250117. doi: 10.1371/journal.pone.0250117 (PMC8101946; doi:10.1371/journal.pone.0250117)
Supplement: S2 File — (PDF) [file pone.0250117.s004.pdf]

Form. NGCT02-2017

Surveyor's Code.....................

QUESTIONNAIRE

Participant's Code	
Date of first evaluation (day, month, year) / Hour: minutes	/
**Socio-epidemiological component**	
Please tell us your date of birth (day, month, year)	Are you part of any FSWs' association?<br>Yes / No
Where were you born? City:	Country: ....
Where do you live? Please only refer to the place where you spend most of the time<br>City: .... Country:	
Do you spend time in another city?	Yes / No
What is the reason why you have to spend time in another city?<br>When was the last time you were in another city because of job?	Job reasons / Study reasons / Family reasons<br>........ weeks ago
How long have you had this job? Please, tell us in years	.... year(s)
**Background**	
Has a physician ever told you if you have chronic pelvic pain syndrome?	Yes / No / I don't know
Has a physician ever told you if you are HIV positive?	Yes / No / I don't know
Has a physician ever told you if you have had Syphilis?	Yes / No / I don't know
Has a physician ever told you if you have had Gonorrhea?	Yes / No / I don't know
Has a physician ever told you if you have had Trichomoniasis?	Yes / No / I don't know
Has a physician ever told you if you have had Chlamydia?	Yes / No / I don't know
**Clinical component**	
Have you had pelvic pain for several weeks or months?	Yes / No
Are you currently having vaginal discharge?	Yes / No
Are you currently having pelvic pain?	Yes / No
Do you feel like it burns in your intimate part, in the vagina?	Yes / No
Do you feel that there are injuries in your intimate part, in the vagina?	Yes / No
Do you feel that there are pimples in your intimate part, in the vagina?	Yes / No
Do you feel any pain when having sexual intercourses?<br>If your answer is Yes, please tell us if the pain is slight, deep, or both	Yes / No<br>Slight / Deep / Both
When was the last time you had vaginal sex without condom with your clients?	.... weeks
When was the last time you had anal sex without condom with your clients?	.....................weeks
When was the last time you had oral sex without condom with your clients?	.... weeks
**Treatment Background**	
When was the last time a doctor treated you for sexually transmitted diseases? (Weeks)	☐ .... weeks ago<br>☐ I haven't received any treatment
What did you receive as treatment?	☐ Antibiotics (PO or IV) ....<br>☐ Ovules ....<br>☐ Creams ....<br>☐ Pain pills ....<br>☐ Not apply
When was the last time you took medication **(without medical prescription)** for vaginal discharge or lower abdominal pain, be it ovules, creams, pills, or injections?	☐ .... weeks ago<br>☐ I haven't taken any treatment
What did you take as treatment?	☐ Antibiotics (PO or IV) ....<br>☐ Ovules ....<br>☐ Creams ....<br>☐ Pain pills ....<br>☐ Not apply
So far this year, how many times have you had a medical examination?	.... times

Surveyor's Code………………..

CUESTIONARIO

Código participante	
Fecha de primera evaluación (día, mes, año) / Hora: minutos	/
**Componente Socio epidemiológico**	
Por favor dígame su fecha de nacimiento (día, mes, año)	¿Pertenece a alguna asociación de MTS? Si / No
¿Dónde nació? Ciudad…………………………………………	País: ………………………………….……
¿En qué lugar vive la mayor parte del tiempo? Ciudad: …………………………………	País: ……………………………
¿Pasa tiempo también en otra ciudad?	Si / No
¿Cuál es el motivo por el que principalmente pasa tiempo en otra ciudad?<br>¿Cuándo fue la última vez que estuvo en otra ciudad por su trabajo?	Por su trabajo / estudios / visita familiar /<br>Hace ……..……………semanas
¿Cuánto tiempo lleva en este trabajo? Por favor dígame en años	………………. año (s)
**Antecedentes**	
Alguna vez le ha dicho un médico si es que tiene síndrome de dolor pélvico crónico?	Si / No / No sabe
Alguna vez le ha dicho un médico si es que tiene VIH positivo?	Si / No / No sabe
Alguna vez le ha dicho un médico si es que ha tenido Sífilis?	Si / No / No sabe
Alguna vez le ha dicho un médico si es que ha tenido Gonorrea?	Si / No / No sabe
Alguna vez le ha dicho un médico si es que ha tenido Tricomonas?	Si / No / No sabe
Alguna vez le ha dicho un médico si es que ha tenido Clamidia?	Si / No / No sabe
**Componente clínico**	
Tiene usted dolor pélvico por varias semanas o meses?	Si / No
Está usted actualmente con secreción vaginal?	Si / No
Siente actualmente dolor pélvico?	Si / No
Siente como que le quema en su parte íntima, en la vagina?	Si / No
Siente que hay lastimados en su parte íntima, en la vagina?	Si / No
Siente que hay granitos en su parte íntima, en la vagina?	Si / No
¿Siente dolor durante las relaciones sexuales?<br>Si responde que "Si", el dolor es: ligero o es muy fuerte y profundo, o ambos?	Si / No<br>Dolor ligero / Dolor profundo / Ambos
¿Cuándo fue la última vez que tuvo relaciones vaginales sin usar condón con sus clientes?	…………………semanas
¿Cuándo fue la última vez que tuvo relaciones anales sin usar condón con sus clientes?	…………………semanas
¿Cuándo fue la última vez que tuvo relaciones orales sin usar condón con sus clientes?	…………………semanas
**Antecedentes de tratamiento**	
¿Cuándo fue la última vez que le dio un médico un tratamiento para enfermedades de transmisión sexual? (Semanas)	☐ Hace ……………………….……. semanas<br>☐ No he recibido tratamiento
¿Qué le dio?	☐ Antibióticos (VO o IV) ………………………..……<br>☐ Óvulos ……………………………………..……….<br>☐ Cremas …………………………………………….<br>☐ Pastillas para el dolor …………………………..<br>☐ No aplica
¿Cuándo fue la última vez que tomó medicamentos **por su cuenta** para la secreción vaginal o para dolor del abdomen inferior, ya sea óvulos, cremas, pastillas o inyecciones?	☐ Hace ……………. semanas<br>☐ No he tomado tratamiento
¿Qué uso?	☐ Antibióticos (VO o IV) ………………………..……<br>☐ Óvulos ………………………………………………<br>☐ Cremas …………………………………………….<br>☐ Pastillas para el dolor …………………………..<br>☐ No aplica
En los que va de este año, ¿cuántas veces se ha hecho examinar para el control?	……………………………. veces
